# P-173. An Exploration of the Reported Cases of *Acanthamoeba* Keratitis

**DOI:** 10.1093/ofid/ofae631.378

**Published:** 2025-01-29

**Authors:** Nazia Nagi, Tahsin Farid

**Affiliations:** CURE ID FDA/NCATs/NIH/CDRC joint Initiative, Department of Infectious Disease/Rare Disease/Rare Cancer, Delhi, Delhi, India; US Food & Drug Administration, Richmond, Texas

## Abstract

**Background:**

Acanthamoeba keratitis (AK) is a rare manifestation of infection caused by this ubiquitous protozoan parasite. Treatment is complicated by the absence of FDA approved treatments. Here we aim to review reported cases of AK to identify case characteristics and potential successful treatments.

WHO Region wise distribution of Acanthamoeba Keratitis

**Figure:**
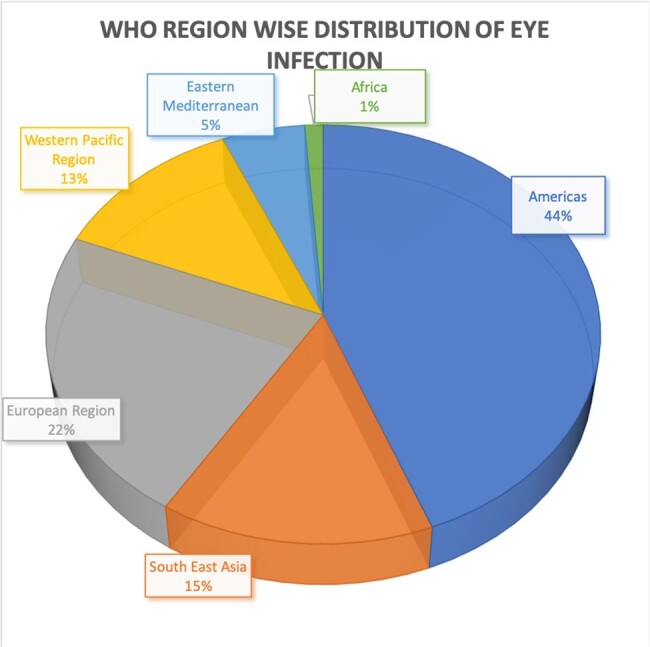

**Methods:**

All cases of AK from 2012-2024 reported on PubMed (684) and Embase (816) were identified using relevant keywords, MeSH terms and Emtree terms. Rayyan.ai was used to screen cases using PRISMA protocol. Cases meeting inclusion criteria were uploaded to CureID. All AK cases in CURE ID (170) were then analyzed in aggregate.

Outcomes in Patients with Acanthamoeba keratitis

**Figure:**
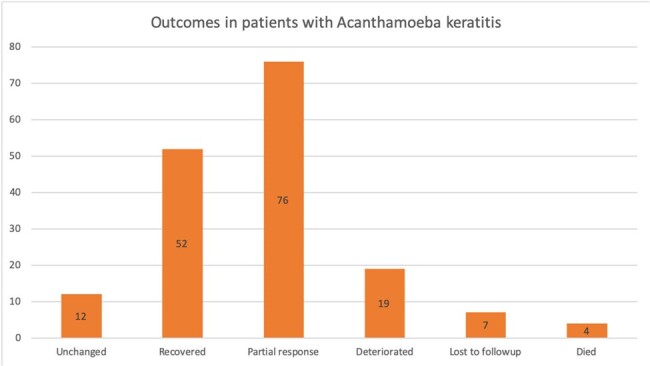

**Results:**

Most cases occurred in the Americas (44.1%), followed by Europe (22.4%) and the least number of cases were contracted in the Eastern Mediterranean (5.3%) region (Figure 1). 76/170 cases of AK showed partial response to the treatment and 12/170 remained unchanged (Figure 2). Table 1 shows the most commonly used medications and their respective outcomes. Figure 4 represents the correlation between type of contact lens used and severity of disease presentation for the 90 cases where this information was available.

Most commonly used medications and their respective outcomes

**Figure:**
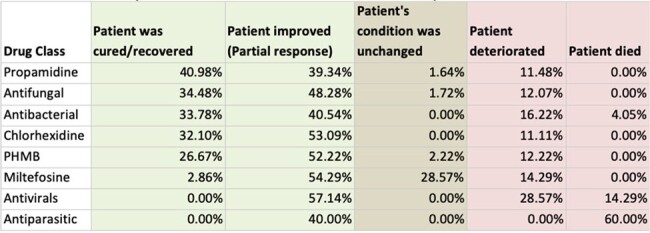

**Conclusion:**

Although majority of the patients responded to treatment, most did not fully recover, and 4 patients died. This underscores the significant morbidity caused by this disease. We noticed diagnostic delays due to misdiagnoses as fungal or viral eye infections. Improved diagnosis will be vital to improving outcomes. Outcomes appeared to be better with combination therapies that included Chlorhexidine, Propamidine and PHMB. This finding supports the inclusion of these drugs in a standardized regimen for treatment of AK. Conversely, Miltefosine produced mixed results and should be excluded. It also appears that severity of AK may be related to contact lens use and soft contact lenses may be associated with higher severity of AK. We speculate that higher incidences in the Americas and Europe may also be related to higher lens usage. Our aim is to further investigate these correlations through a full manuscript.

Lens use V/S No Lens use and severity in presenting symptoms

**Figure:**
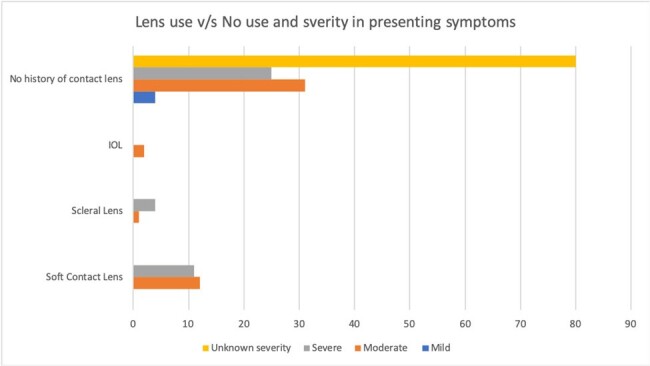

**Disclosures:**

**All Authors**: No reported disclosures

